# Preparation and In Vitro Evaluation of RITUXfab-Decorated Lipoplexes to Improve Delivery of siRNA Targeting C1858T PTPN22 Variant in B Lymphocytes

**DOI:** 10.3390/ijms23010408

**Published:** 2021-12-30

**Authors:** Andrea Arena, Eugenia Belcastro, Antonella Accardo, Annamaria Sandomenico, Olivia Pagliarosi, Elisabetta Rosa, Stefania Petrini, Libenzio Adrian Conti, Ezio Giorda, Tiziana Corsetti, Riccardo Schiaffini, Giancarlo Morelli, Alessandra Fierabracci

**Affiliations:** 1 Infectivology and Clinical Trials Research Department, Bambino Gesù Children’s Hospital, Scientific Institute for Research, Hospitalization and Healthcare (IRCCS), 00146 Rome, Italy; aarena026@gmail.com (A.A.); eugenia.belcastro@opbg.net (E.B.); olivia.pagliarosi@opbg.net (O.P.); 2 Research Centre on Bioactive Peptides (CIRPeB), Department of Pharmacy, University of Naples Federico II, 80134 Naples, Italy; antonella.accardo@unina.it (A.A.); elisabetta.rosa@unina.it (E.R.); gmorelli@unina.it (G.M.); 3 Institute of Biostructures and Bioimaging (IBB), National Research Council (CNR), 80134 Naples, Italy; sandomenico@cnr.it; 4 Confocal Microscopy Core Facility, Research Laboratories, Bambino Gesù Children’s Hospital, Scientific Institute for Research, Hospitalization and Healthcare (IRCCS), 00146 Rome, Italy; stefania.petrini@opbg.net (S.P.); libenzioadrian.conti@opbg.net (L.A.C.); 5 Research Laboratories, Bambino Gesù Children’s Hospital, Scientific Institute for Research, Hospitalization and Healthcare (IRCCS), 00146 Rome, Italy; ezio.giorda@opbg.net; 6 Unit of Hospital Pharmacy, Bambino Gesù Children’s Hospital, Scientific Institute for Research, Hospitalization and Healthcare (IRCCS), 00165 Rome, Italy; tiziana.corsetti@opbg.net; 7 Diabetes and Growth Pathology Unit, Bambino Gesù Children’s Hospital, Scientific Institute for Research, Hospitalization and Healthcare (IRCCS), 00165 Rome, Italy; riccardo.schiaffini@opbg.net

**Keywords:** T1D, autoimmune disease, functionalized lipoplexes, rituximab, immunotherapy, variant PTPN22

## Abstract

Autoimmune endocrine disorders, such as type 1 diabetes (T1D) and thyroiditis, at present are treated with only hormone replacement therapy. This emphasizes the need to identify personalized effective immunotherapeutic strategies targeting T and B lymphocytes. Among the genetic variants associated with several autoimmune disorders, the C1858T polymorphism of the protein tyrosine phosphatase non-receptor type 22 (*PTPN22*) gene, encoding for Lyp variant R620W, affects the innate and adaptive immunity. We previously exploited a novel personalized immunotherapeutic approach based on siRNA delivered by liposomes (lipoplexes) that selectively inhibit variant allele expression. In this manuscript, we improved lipoplexes carrying siRNA for variant C1858T by functionalizing them with Fab of Rituximab antibody (Ritux_Fab_-Lipoplex) to specifically target B lymphocytes in autoimmune conditions, such as T1D. Ritux_Fab_-Lipoplexes specifically bind to B lymphocytes of the human Raji cell line and of human PBMC of healthy donors. Ritux_Fab_-Lipoplexes have impact on the function of B lymphocytes of T1D patients upon CpG stimulation showing a higher inhibitory effect on total cell proliferation and IgM+ plasma cell differentiation than the not functionalized ones. These results might open new pathways of applicability of Ritux_Fab_-Lipoplexes, such as personalized immunotherapy, to other autoimmune disorders, where B lymphocytes are the prevalent pathogenic immunocytes.

## 1. Introduction

Autoimmune diseases encompass a broad category of tissue-targeted conditions including organ-specific ones, often affecting endocrine glands like insulin-dependent diabetes mellitus (type 1 diabetes, T1D) and thyroiditis, and non-organ specific ones like systemic lupus erythematosus (SLE), vasculitides, rheumatoid arthritis (RA) and systemic sclerosis (SS) [1]. The incidence of autoimmune diseases is increasing worldwide [2]. With reference to endocrine autoimmunity and T1D, the latter especially occurs in children below the age of five [3]. It is generally recognized that autoimmunity is caused by autoreactive effector cells, that is, autoreactive T lymphocytes for T1D whilst autoreactive B lymphocytes play a major role in SLE. Pathogenic T cell precursors result from the defective process of immunological tolerance that takes place in the thymus in perinatal age under the influence of genetic susceptibility and epigenetic modifications and it is triggered by putative exogenous and endogenous environmental agents (i.e., the microbiome) [4,5]. Nevertheless, the onset of clinical disease is caused by still unknown eliciting antigens that may be different from those which generate the tolerance breakdown and act after a long preclinical period.

Complexity and heterogeneity are the hallmarks of this category of disorders; therefore, this emphasizes the need to identify more effective strategies for their personalized prevention and treatment. At present, the only available treatment for endocrine organ-specific autoimmune disorders is the substitutive administration of the deficient hormone. Therefore, a theoretical advance is the contribution that an immunotherapeutic strategy, by halting the pathogenetic mechanism of disease, may add to preserve the hormonal cells from the autoimmune attack of autoreactive T cells [6].

Several approaches targeting the immune system have already been experimented with, but without success, especially for the treatment of T1D since insulin-independence was not achieved in diabetic patients [7,8]. Indeed, these approaches in particular explored the possibility to target T lymphocytes, the main pathogenic effectors.

B lymphocytes are also important targets in autoimmunity because they give rise to autoantibody-producing plasma cells and induce CD4+ T cell differentiation by antigen presentation. Moreover, they regulate the organization of the lymphoid system architecture and play a role in co-stimulation, controlling the function of dendritic cells and the secretion of soluble factors, that is, proinflammatory cytokines tumor necrosis factor alpha (TNF-α), interleukin (IL)-8 and IL-6 [9]. Therefore, B cell modulating therapies in B cell-mediated autoimmune diseases were attempted in order to maintain immune surveillance, eliminate effector B lymphocytes and stimulate regulatory B cells (B-regs) [10]. These strategies have employed Rituximab [11] and anti-CD20 antibodies of a new generation, that is, anti-CD19, -CD22, chimeric autoantibody receptor T (CAAR-T) cells and inhibitors of B cell receptor activation [9]. With variable applications to specific autoimmune conditions, these treatments had limited efficacy or generated adverse effects since they also affected the B-regs cellular subset or halted the humoral antigen recall response thus enhancing risk [12].

In light of the foregoing, tailored approaches of ‘personalized medicine’ should be attempted especially on the basis of disease susceptibility under genetic influence. With the advent of genome-wide linkage, candidate gene and genome-wide association studies [13], in addition to human leucocytes antigens, several single nucleotide polymorphisms (SNPs) were discovered to contribute to autoimmunity etiopathogenesis [14,15]. In citing examples of candidate common susceptibility genes involved in immune regulation, cytotoxic T lymphocyte-associated antigen 4 (CTLA4) suppresses T cell activation [16], forkhead box P3 (FOXP3) is involved in the differentiation of T regulatory cells (T-regs), the IL-2 receptor alpha/CD25 gene also affects the development and the function of T-regs, and the TNF-α gene, located on chromosome 6p21.3, is at the basis of the increased risk for the association of T1D and autoimmune thyroid disease (rev. in [15]). Among the others, protein tyrosine phosphatase non-receptor type 22 (*PTPN22*), encoding for the lymphoid tyrosine phosphatase (Lyp), affects the TCR signaling pathway. The C1858T polymorphism of *PTPN22*, which replaces the amino acid Arg (R) 620 with Trp (W) (R620W), encodes for a more active phosphatase, namely Lyp variant R620W. This is a potent inhibitor of T cell activation involved in several autoimmune diseases [17,18]. The variant is supposed to have important effects at the level of thymocyte tolerization [18]. In particular, the C1858T allelic variant represents a genetic risk factor for T1D [17,19,20,21], Graves’ disease [22], the association of T1D and autoimmune thyroid disease known as autoimmune polyglandular syndrome type 3 variant (APS3v) (rev. in [6]) and myasthenia gravis [23]. Even for non-organ specific autoimmune diseases of complex pathogenesis, the association with the *PTPN22* polymorphism was observed in Caucasians, that is, SLE (rev. in [20,24]), Wegener’s granulomatosis [25] and RA patients [18,26].

We provided evidence that the C1858T *PTPN22* gene polymorphism [6,18] could be a relevant target for immunomodulation in the treatment of C1858T patients affected by an autoimmune disease, that is, T1D [6,27]. In particular, we demonstrated the possibility of achieving target down-modulation of variant C1858T *PTPN22* gene by delivering siRNA molecules with liposomes to peripheral blood mononuclear cells (PBMC) in culture [6]. Strategies can be implemented to improve the targeted delivery of lipoplexes to specific immunotypes playing a major role in the autoimmune disease pathogenesis. On a theoretical basis, this can be unraveled by using monoclonal antibodies that drive nanoparticles to T or B lymphocytes through Food and Drug Administration (FDA) approved humanized monoclonal antibodies (MoAbs) [28,29,30]. In light of the foregoing, the aim of this manuscript was to evaluate the possibility of generating functionalized lipoplexes with Fab of anti-CD20 (Rituximab) [30] in order to target B lymphocytes in autoimmune diseases.

## 2. Results

### 2.1. Preparation of Proteolytic Fab’ Fragment

**Figure 1:**
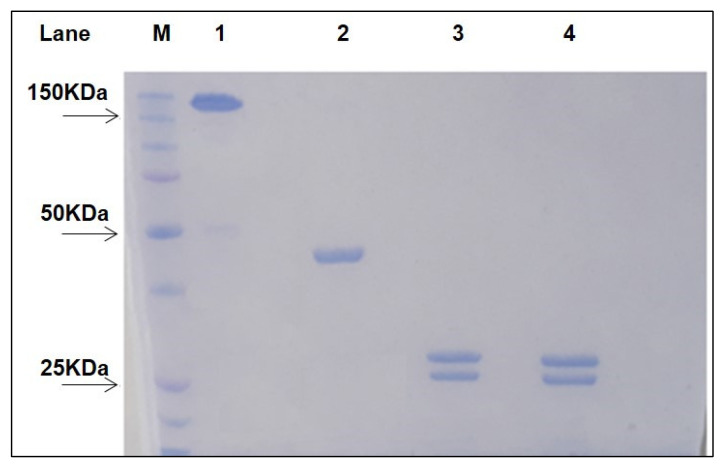
SDS-PAGE of purified Ritux_Fab2_ and Ritux_Fab._ The two samples were loaded under non-reducing and reducing (lanes 3–4) conditions, respectively. Lane 1: non reduced Ritux_Fab2_; Lane 2: non reduced Ritux_Fab_; Lane 3: reduced Ritux_Fab2_; Lane 4: reduced Ritux_Fab_; Lane M: Protein Precision Blue MW Marker (Biorad) used as reference.

Fragment antigen binding (Fab) of Rituximab (Ritux_Fab_) was prepared by reduction of the F(ab’)_2_ of Rituximab (Ritux_Fab2_) obtained by a standard proteolytic cleavage with pepsin. Analytical size exclusion chromatography (SEC)-HPLC showed that both Ritux_Fab2_ and Ritux_Fab_ were purified to homogeneity (purity over 95%, Supplementary Figure S1A,B). Sodium dodecyl sulphate–polyacrylamide gel electrophoresis (SDS-PAGE) analysis showed that Ritux_Fab2_ and Ritux_Fab_ had an apparent molecular mass of ~150 kDa and ~50 kDa in native conditions, respectively (Figure 1).

**Figure 2:**
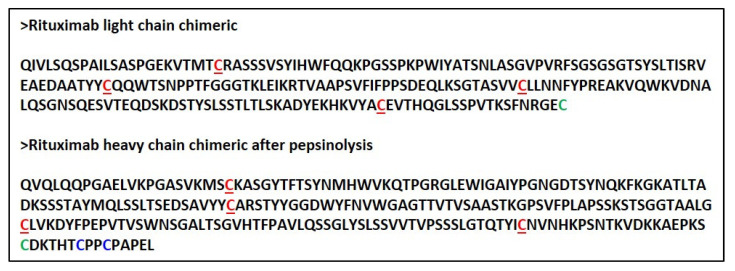
Aminoacidic sequence of intact LC and HC of Rituximab as cut following pepsin hydrolysis. Cysteine involved in intrachain disulfide bridges are underlined and reported in red. C224 of the HC and C213 of the LC that connect the chains are reported in bold green. The free cysteines present in the Fab’ hinge region in the HC are in bold blue.

Upon reduction, the products were stained as two bands at about 25 kDa and 23 kDa, corresponding to the heavy (HC) and light chains (LC), respectively. The sequences of the LC and HC (residues 1–238) of Rituximab are reported in Figure 2 (https://go.drugbank.com/drugs/DB00073, accessed on 10 November 2021). The HC was cut by pepsin at the C-terminus of L238 leaving, after the reduction of F(ab)_2_, cysteine 230 and cysteine 233 side chains as free thiols. Ritux_Fab2_ and Ritux_Fab_ were analyzed by liquid chromatography coupled with electrospray ionization ion-traptime-of-flight mass spectrometry (LC-ESI-TOF-MS) after extensive reduction. The chromatogram showed the two distinct chains eluted at different retention times according to their hydrophobicity (LC and HC eluted at 13.9 and 14.8 min (min), respectively) (Supplementary Figure S2A). Deconvolution of mass/charge spectra of the separated chains revealed that both experimental molecular weights (MWs) were in agreement with those calculated (average MW), considering the partial reformation of the intradomain disulphide bridges. For the LC, an experimental MW of 23,035.82 Da (Supplementary Figure S2B), which was comparable to the calculated value of 23,038.33 Da, was observed. The Δmass of −2.51 Da suggested that the two intramolecular bridges were partially reformed. For the HC, an experimental MW of 25,154.86 Da (Supplementary Figure S2C), which is comparable to the calculated value of 25,175.53 Da (Δmass = −20.67 Da), was observed. In this case, the N-terminal glutamine was fully converted to pyroglutamic acid (Δmass = −17.03 Da) and the two intramolecular bridges were almost fully reformed (Δmass = −4.0 Da). Considering these modifications, the calculated MW was 25,154.50 Da and the Δmass with the experimental value was only = −0.36 Da. In order to confirm the occurrence of free cysteines in the hinge region of the HC a reaction of alkylation with 4-[(isopropylaminomethyl] phenylamine (IAM) (Δmass = +57.02 Da) was also performed on the Ritux_Fab_ in native conditions and the MW was again evaluated by LC-ESI-TOF analysis under reducing conditions. The HC was detected as three main species corresponding to the polypeptide modified with one, two or three IAMs, with the double modified chain being the prevailing species. The MWs of the three species were: HC+1 IAM, experimental MW 25,211.06 Da, calculated MW 25,211.50 Da; HC+2 IAM, experimental MW 25,268.54 Da, calculated MW 25,268.52 Da; HC+3 IAM, experimental MW 25,325.33 Da, calculated MW 25,325.52 Da. The presence of the triple modified HC was likely due to an alkylation occurring after reduction. The fluorescein isothiocyanate (FITC)-labelled Ritux_Fab_ was also characterized by LC-ESI-TOF analysis under reducing conditions. The MW of the LC was detected as two main species corresponding to the unmodified polypeptide (experimental MW 23,035.75 Da) and to the single modified product (experimental MW 23,425.60 Da, Δmass = +389.38 Da). The HC was identified as three main species corresponding to the unlabeled HC (experimental MW 25,155.25 Da), and to the single and double FITC-modified HC (experimental MWs: 25,544.42 Da, Δmass = +389.38 Da; 25,933.81 Da, Δmass = +778.56 Da).

### 2.2. Circular Dichroism (CD) Structural Characterization

**Figure 3:**
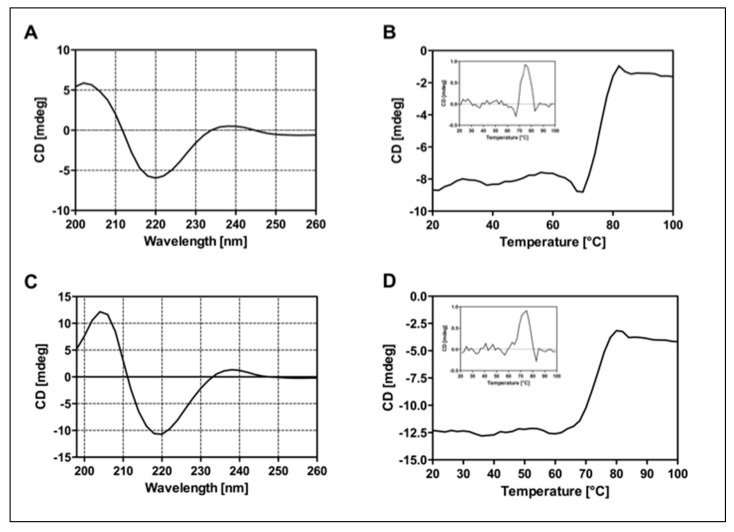
Panel of CD analyses. Far-UV spectra of Ritux_Fab2_ (**A**) and Ritux_Fab_ (**C**) recorded at the concentration of 4 µM at 20 °C. Denaturation curves were collected between 20 °C and 95 °C monitoring the CD signal at 218 nm. Ritux_Fab 2_ (**B**) and Ritux_Fab_ (**D**).

CD analysis was performed on Ritux_Fab2_ and Ritux_Fab_ fragments to evaluate proper folding in terms of secondary structure and stability. CD spectra recorded in the far-UV region (Figure 3A–C) showed that fragments adopted a classical β-sheet secondary structure according to the Ig-like folding, with a negative band at 219 nm and a positive one at 205 nm. Thermal denaturation performed by monitoring the CD signal at 218 nm from 20 °C to 95 °C revealed a melting temperature (Tm) of 75 °C for Ritux_Fab2_ and 74 °C for Ritux_Fab_ (Figure 3B–D). The data suggested that the proteolytic fragments retain a correct folding and stability comparable to that of the whole antibody.

### 2.3. Lipoplex Formulation and DLS Characterization

**Figure 4:**
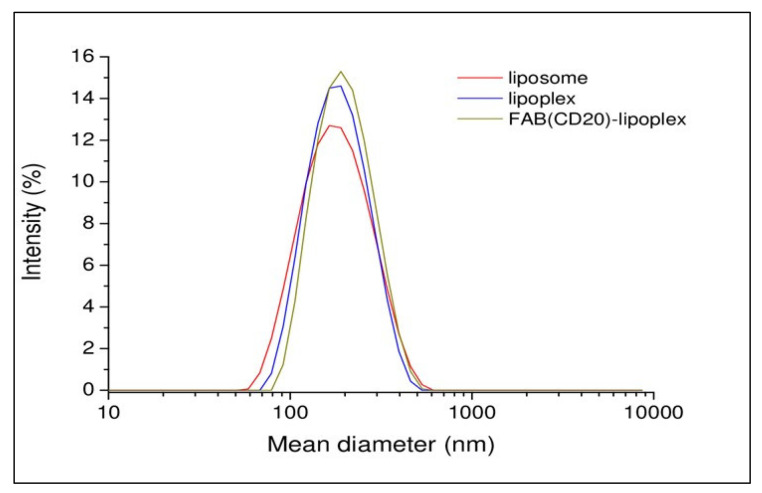
Intensity profiles of liposomal formulations.

Cationic liposomes were prepared using commercial phospholipid, 1,2-Dioleoyl-sn-glycero-3-phosphoethanolamine (DOPE), N-[1-(2,3-Dioleoyloxy)propyl]-N,N,N-trimethylammonium chloride (DOTAP), 1,2-distearoyl-sn-glycero-3-phosphoethanolamine-N-[maleimide(polyethylene glycol)-2000] (ammonium salt) (DSPE-PEG2000-Maleimide) (DOPE/DOTAP/DSPE-PEG2000-Maleimide) in 47.5/47.5/5 molar ratio. Briefly, the lipidic film prepared in chloroform was hydrated in phosphate buffer saline (PBS) at pH 7.4. Then, the suspension was sonicated for 30 min and extruded 10-times through a 0.1 μm polycarbonate membrane. Lipoplexes were prepared incubating the liposomal solution with siRNA at room temperature (RT) for 3 h (h). In this preparation, the amount of siRNA was 3.2 μM. Targeted Ritux_Fab_-Lipoplexes were obtained by functionalizing the lipoplex with Ritux_Fab._ The coupling reaction was obtained by reacting overnight (ON), under nitrogen, the Fab’ thiol function with the maleimide group, according to the Michael addition reaction. Functionalized lipoplex were purified from free Fab’ by gel filtration. All the aggregates were structurally characterized by dynamic light scattering (DLS) technique. Intensity profiles of liposomal preparation are reported in Figure 4. From the inspection of the figure, it can be observed that all the distributions are monomodal with one slow diffusion mode around 150 nm. Moreover, no significant changes in the size of aggregates were observed after functionalization of the lipoplex surface with Fab’. Fluorescent lipoplexes were obtained by inserting lipophilic dye (such as Rho-PE) in the liposome formulation and functionalizing the liposomal surface with a small amount of Ritux_Fab_-FITC.

### 2.4. Ritux_Fab_ and Liposomes Functionalized with Ritux_Fab_ Specifically Bind to Raji Cells

**Figure 5:**
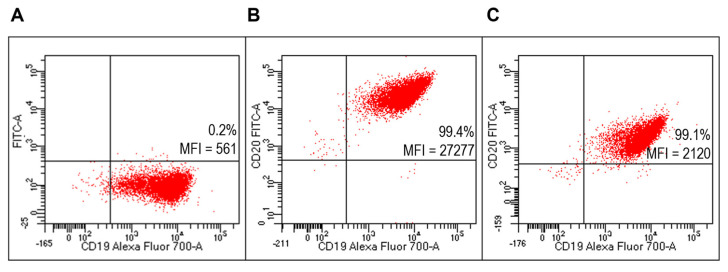
Evaluation of FITC-Ritux_Fab_ functionalized liposomes on Raji cells (B-cell lymphoma line). Representative FACS profiles indicating the MFI values of FITC-Ritux_Fab_ among Raji cells. Cells were stained with the anti-CD19-Alexa Fluor 700 (**A**) and FITC-Ritux_Fab_ (**B**) or liposomes functionalized with Ritux_Fab_/FITC-Ritux_Fab_ (90/10 mol/mol) (**C**).

Flow cytometry analysis (FACS) detected specific reactivity FITC-Ritux_Fab_ to B lymphocytes of the Raji cell line (human B lymphocyte; Burkitt’s lymphoma) as revealed by mean fluorescence intensity (MFI) values (Figure 5B). Further, the mixture composed of liposomes functionalized with Ritux_Fab_/FITC-Ritux_Fab_ (90/10 mol/mol) binded efficiently and with high specificity to cells of the B lymphoma Raji cell line (Figure 5C, Supplementary Figure S3).

### 2.5. Ritux_Fab_ and Liposomes Functionalized with Ritux_Fab_ Specifically Bind to B Lymphocytes within Human PBMC

**Figure 6:**
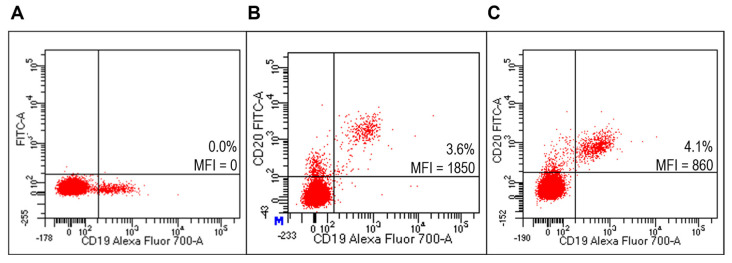
Evaluation of liposomes derivatized with FITC-Ritux_Fab_ on HD PBMC. Representative FACS profiles indicating the MFI values of FITC-Ritux_Fab_ labelled among HD PBMC. Cells were stained with the anti-CD19-Alexa Fluor700 (**A**) and FITC-Ritux_Fab_ (**B**) or combination of liposomes functionalized with Ritux_Fab_/FITC-Ritux_Fab_ (90/10 mol/mol) (**C**).

**Figure 7:**
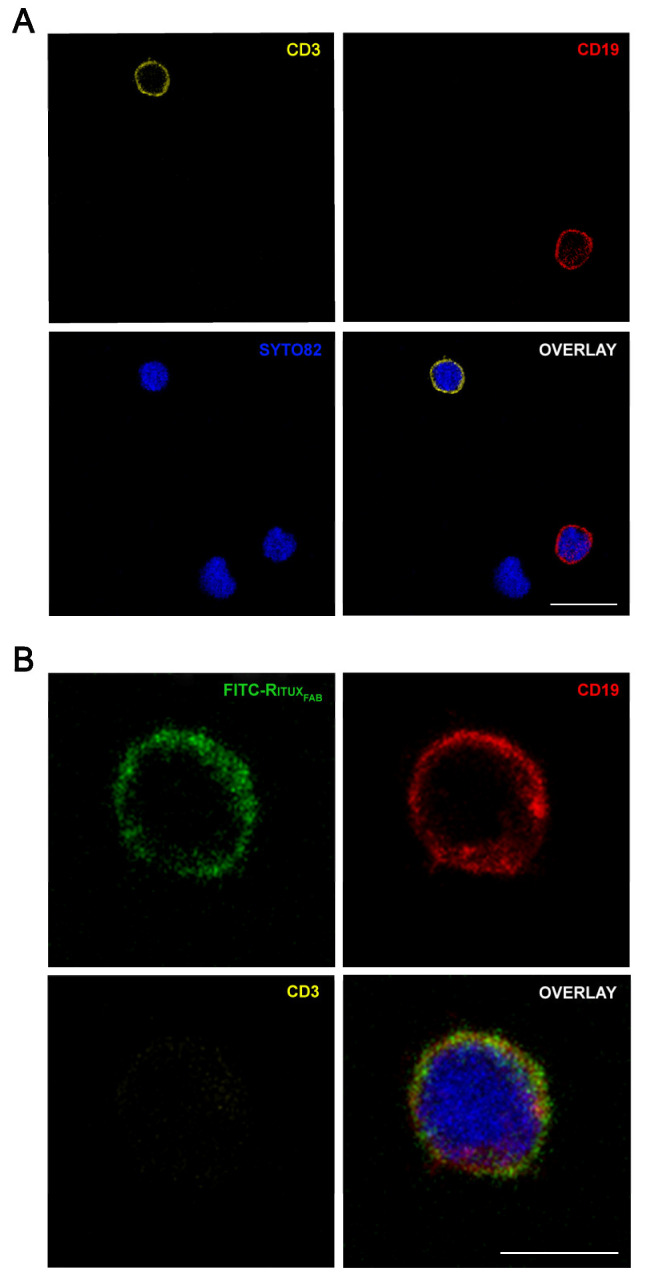
Confocal microscopy analysis of FITC-Ritux_Fab_ in HD PBMC. Images show the expression of CD3+ (pseudocolored in orange, upper left panel) and CD19+ (red, upper right panel) cells (**A**) and the presence of FITC-Ritux_Fab_ (green, upper left panel) in CD19+ (red, upper right panel) cells and the simultaneous absence in CD3+ (orange, lower left panel) (**B**) among lymphocytes within the human PBMC pool from HD. Cell nuclei are counterstained with SYTO™82 dye (pseudocolored in blue). Scale bars = 10 μm (**A**) and 5 μm (**B**).

FACS analysis detected specific reactivity of FITC-Ritux_Fab_ to B lymphocytes within the human PBMC pool from healthy donors (HD) (Figure 6B, Supplementary Figure S4). Further, the mixture composed of liposomes functionalized with Ritux_Fab_/FITC-Ritux_Fab_ (90/10 mol/mol) binded efficiently and with high specificity to B lymphocytes within the same pool (Figure 6C). Reactivity of FITC-Ritux_Fab_ was also revealed on B lymphocytes by confocal microscopy analysis (Figure 7).

### 2.6. Targeted Lipoplexes Specifically Bind to Human B Lymphocytes

**Figure 8:**
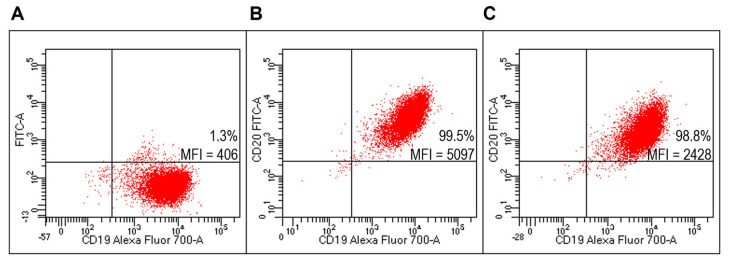
Evaluation of targeted lipoplexes on Raji cell line. Representative FACS profiles indicating the percentages of Fab(CD20)-FITC+ among Raji cells after staining with unlabeled and untargeted lipoplex (**A**), FITC-Ritux_Fab_ (**B**) or targeted lipoplex (**C**).

**Figure 9:**
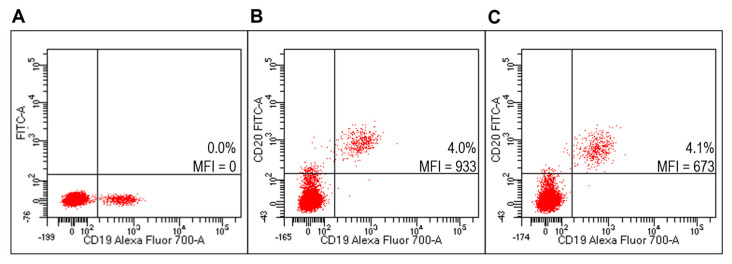
Evaluation of targeted lipoplexes on HD PBMC. Representative FACS profiles indicating the percentages of Fab(CD20)-FITC+ among lymphocytes after staining with unlabeled and untargeted lipoplex (**A**), FITC-Ritux_Fab_ (**B**) or FITC-Ritux_Fab_ lipoplexes (**C**).

Lipoplexes functionalized with FITC-Ritux_Fab_ specifically binded to Raji cells (Figure 8C) compared to unlabeled lipoplex (Figure 8A). They also specifically binded to B lymphocytes within the human HD PBMC pool (Figure 9C).

By using targeted lipoplex labeled with 1,2-dioleoyl-sn-glycero-3-phosphoethanolamine-N-(7-nitro-2-1,3-benzoxadiazol-4-yl) (ammonium salt) (NBD-PE) fluorophore, we verified their specific MFI fold increase of binding to CD19+ cells on the Raji human cell line (Supplementary Figure S5) and on B lymphocytes within HD PBMC (Supplementary Figure S6) than using the corresponding untargeted lipoplexes.

### 2.7. Effect of Functionalized Lipoplexes on CpG—Stimulated B Lymphocytes within Human PBMC

**Figure 10:**
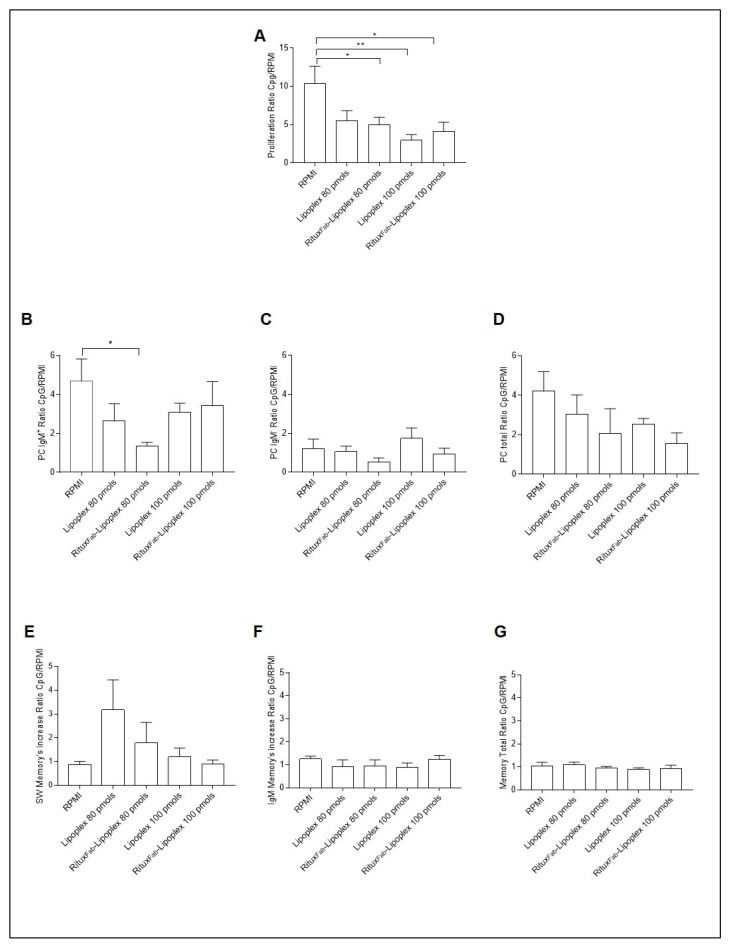
B cell phenotype after 4 days of CpG stimulation in T1D patients carrying the C1858T *PTPN22* variant after treatment with lipoplex and Ritux_Fab_-Lipoplex at different doses. Ratio of proliferation of CpG stimulated over unstimulated CMFDA-labelled CD19+ cells (**A**), ratio of plasma cells from IgM+ (**B**), IgM− (**C**) and total (**D**) memory B cells. Ratio of switched memory (SW) B cell (**E**), IgM+ memory B cells (**F**) and total memory B cells (**G**). Data are expressed as mean ± SEM of *n* = 5. * *p* < 0.05, ** *p* < 0.01.

The B cell phenotypes of the PBMC from T1D patients carrying the C1858T *PTPN22* variant after treatment of targeted and untargeted lipoplexes at 80 and 100 pmols of siRNA were analyzed after 4 days of CpG oligodeoxynucleotides stimulus (Figure 10). A significant decrease in the proliferative response (calculated as ratio of proliferation of CD19+ cells over unstimulated cells (RPMI)) was observed with targeted lipoplexes with both doses (Figure 10A).

Regarding the specific immunophenotype, the ratio of plasma cells from IgM+ memory B cells showed a higher inhibitory effect with lipoplex at the respective concentration (Figure 10B).

## 3. Discussion

In this manuscript, we provided evidence of the feasibility of exposing Fab of a MoAb, that is, Rituximab on lipoplexes delivering siRNA against the C1858T *PTPN22* variant. Indeed, functionalized lipoplexes bound themselves with high specificity to B lymphocytes of the human Raji cell line and within the PBMC pool. Remarkably, as revealed by FACS analysis, Ritux_Fab_ -functionalized lipoplexes exhibited a higher fold increase of binding to B lymphocytes of the human Raji cell line and of PBMC than not functionalized lipoplexes. This evidence suggests that functionalization does not affect the biofunctionality of Ritux_Fab_.

To complete the functional evaluation of Ritux_Fab_-Lipoplexes, we also unraveled whether they could have a significant effect on the function of B lymphocytes with respect to unfunctionalized lipoplex in comparison to lipoplex following CpG stimulation in PBMC of an exemplary autoimmune disease, that is, T1D. Indeed, we previously observed altered B cell homeostasis and Toll-Like receptor 9-driven response in T1D carriers of the *PTPN22* C1858T allelic variant [31,32]. Data demonstrated that the IgM+ memory B cells were significantly increased in heterozygous (1858C/1858T) T1D patients compared to C/C individuals and in the groups of individuals who were heterozygous for the variant compared to C/C individuals [31]. As regards the functionalization of nanosystems like polymeric micelles and liposomes with antibodies [33] or other homing entities [34] has been widely described as a suitable strategy to achieve an “active targeting” towards specific tissues/cells overexpressing receptors, thus avoiding side effects on healthy tissues [35]. Indeed, we could demonstrate a significant higher inhibitory effect of Ritux_Fab_-Lipoplexes than not functionalized ones on total B lymphocytes proliferation and IgM+ plasma cell differentiation.

The results of this investigation open future pathways to improving the specific delivery and effect of lipoplexes on immunocytes that play a pathogenetic role in different autoimmune conditions. From this initial evidence, certainly more advanced procedures could be exploited in the future to foster functionalization of lipoplexes by exposing Fab of Rituximab to increase B lymphocytes binding and consequently increase functional effect. This investigation also opens the pathway to functionalized lipoplexes with other FDA approved MoAb including anti-CD3 Teplixumab or Otelixizumab [28,29] for specific targeting of T lymphocytes in T cell-mediated diseases such as T1D.

Additional FDA-approved MoAb could be theoretically used for lipoplexes functionalization for their specific targeting delivery. As regards the integrin heterodimer leukocyte function, associated antigen-1 (LFA-1, CD11a) is a classic adhesion molecule that facilitates T and antigen-presenting cells interaction as well as functioning in activation and trafficking of leukocytes [36]. Antagonism of LFA-1 with MoAb, either alone or in combination with other agents, can induce regulatory tolerance in vivo. Efalizumab, a new generation humanized anti-LFA-1 MoAb, offers at present some promises for clinical application in immunotherapy [37]. LFA-3 (CD58) is a receptor found on the membrane of nearly all human cells. Its interaction with the counter-receptor CD2 generates effective cell–cell adhesion and it is responsible for a costimulatory signal to enhance T lymphocytes responses or modulate anergy/apoptosis. This evidence suggests that it may have therapeutic applications [38]. Alefacept is a chimeric fusion protein composed of CD2-binding portion of LFA3 linked to the Fc region of human IgG1 (LFA3-Fc). Alefacept MoAb was designed to inhibit the activation of memory T lymphocytes that contribute to chronic inflammation in psoriasis [39].

In conclusion, the present findings indicate the efficiency of functionalized lipoplexes with Fab anti-CD20 (Rituximab) to specifically target B lymphocytes in autoimmune conditions, such as T1D. These results propel future investigations of the applicability of Ritux_Fab_ functionalized lipoplexes, such as personalized immunotherapy, to other autoimmune disorders, where B lymphocytes are the prevalent pathogenic immunocytes such as SLE.

## 4. Materials and Methods

### 4.1. Preparation of Rituximab F(ab’)2 and Fab’

Ritux_Fab2_ (CD20, ~100 kDa) was prepared by treatment with pepsin (porcine gastric mucosa pepsin, Sigma-Aldrich, Milan, Italy) using a 20/1 antibody/pepsin ratio (*w*/*w*). The reaction was performed in 100 mM acetate buffer pH 4.0, containing 10 mM ethylenediaminetetraacetic acid (EDTA). After incubation for 16 h at 37 °C, the mixture was purified by SEC on a Superdex S200 column using PBS solution (Sigma-Aldrich) as running buffer [40]. Ritux_Fab2_ was efficiently reduced to Fab’ (Ritux_Fab_, ~50 kDa) by treatment for 2 h at 37 °C with 10 mM β-mercaptoethylamine (MEA) in presence of 2 mM EDTA. After reduction, Ritux_Fab_ was again purified by SEC on a Superdex S200 gel filtration column in PBS buffer. Fractions containing Ritux_Fab2_ and Ritux_Fab_ were analyzed by 12% SDS-PAGE under native and reducing conditions. The concentrations were determined spectrophotometrically on a Nanodrop 2000C instrument (ThermoFisher instruments, Milan, Italy) using a ε_280nm_= 166,060 M^−1^ cm^−1^ for Ritux_Fab2_ and a ε_280nm_= 83,030 M^−1^ cm^−1^ for Ritux_Fab_.

### 4.2. Antibody Fragments Labelling with FITC

Ritux_Fab2_ was randomly labeled with FITC (Sigma Aldrich) by reacting the protein with 10-fold excess FITC in 50 mM NaHCO_3_ pH 8.0 for 1 h at RT. The product, named FITC-Ritux_Fab2_, was extensively dialyzed against PBS buffer at 4 °C, reduced with MEA to obtain FITC-Ritux_Fab_ and then purified by SEC on a Superdex S200 gel filtration column. The concentration of FITC-Ritux_Fab_ was determined spectrophotometrically on a Nanodrop 2000C instrument using a ε_495nm_ = 70,000 M^−1^ cm^−1^. The presence of FITC was also evaluated by LC-ESI-TOF-MS analyses.

### 4.3. Analytical Characterization of Ritux_Fab2_ and Ritux_Fab_

Analytical SEC analyses were performed using a Biosep-SEC 2000 column (300 mm × 7.80 mm Phenomenex) in 50 mM phosphate 100 mM NaCl pH 7.5 applying a flow rate of 0.6 mL/min. UV detection was performed at 215 nm. After reduction and alkylation, the products were identified by LC-ESI-TOF analyses using a C4 BioBasic column (50 mm × 2.1 mm, ThermoFisher Scientific, Monza, Italy) connected to a LC-ESI-TOF MS system from Agilent (Cernusco sul Naviglio, Italy). Separations were obtained at 0.2 mL/min using as solvent A 0.05% *v*/*v* trifluoroacetic acid (TFA) in water and as solvent B 0.05% *v*/*v* TFA in acetonitrile applying a linear gradient from 25% to 65% solvent B in 20 min. The mass analyzer was set to operate in positive ion scan mode with mass scanning from 100 to 3200 *m*/*z*. The ion source was upgraded from the original Agilent Jet Stream (AJS) source to the dual-sprayer version for improved reference mass delivery. Nitrogen was used as drying and nebulizer gas. The instrument acquired data using the following parameters: drying gas temperature, 325 °C; drying gas flow, 10 L/min; nebulizer, 20 psi (pound per square inch) sheath gas temperature, 400 °C; sheath gas flow, 11 L/min; VCap. 3500 V; nozzle, 0 V; fragmentor, 200 V, skimmer, 65 V and octapole RF Vpp was 750. The instrument was set to extended dynamic range mode (2 GHz). Tuning and calibration were performed before sample running. Data collection and integration were performed using MassHunter workstation software (version B.05.00, Agilent Technologies, Santa Clara, CA, USA). Data were stored in both centroid and profile formats during acquisition. A constant flow of Agilent TOF reference solution through the reference nebulizer allowed the system to continuously correct for mass drift by using two independent reference lock-mass ions, purine (*m*/*z* 119.03632) and HP-922 (*m/z* 922.000725), to ensure mass accuracy and reproducibility. Target compounds were detected, and the data analyzed and reported using the Agilent MassHunter Qualitative software (Agilent Technologies, Santa Clara, CA, USA).

### 4.4. Structural Characterization of Ritux_Fab2_ and Ritux_Fab_ by CD

CD spectra were recorded on Ritux_Fab2_ and Ritux_Fab_ using a JASCO J-810 spectropolarimeter (Jasco, Cremella, Italy), equipped with a thermostated cell holder (Peltier system) to change the temperature in a controlled way and interfaced with a Neslab RTE-110 water bath. Far-UV CD spectra were recorded in quartz cuvettes 110-QS with 1.0 mm optical path length (Hellma; Mullheim, Baden, Germany) at a concentration of about 4 μM in 20 mM PBS, pH 7.5, at 20 °C. Spectra were collected in the wavelength range between 198 and 280 nm at a scanning speed of 50 nm/min, with a data pitch of 0.2 nm, a band width of 1 nm, a response of 4 s (sec) and a standard sensitivity. Thermal denaturation experiments were performed by monitoring changes in ellipticity at 218 nm by exposure to increasing temperatures between 20 °C and 95 °C, while heating at 60 °C/h (1 °C/min). Melting points were determined by the method of the first derivative. Four independent spectra for each sample were recorded, averaged and smoothed using the Spectra Manager software, version 1.53. Final spectra were corrected by subtracting the corresponding baseline spectrum obtained under identical conditions.

### 4.5. Liposome Formulation

DOPE, DOTAP, DSPE-PEG2000-Maleimide and NBD-PE were purchased from Avanti Polar Lipids (Alabaster, AL, USA).

All solutions were prepared by weight and buffered at pH 7.4 using 100 mM PBS and the pH was controlled using pH meter MeterLab PHM220. Mixed liposomes DOTAP/DOPE/DSPEPEG2000-Maleimeide (47.5/47.5/5 molar ratio) were prepared by dissolving the required amounts of phospholipids in 1 mL of chloroform. Subsequently, the organic solvent was removed under a stream of nitrogen gas to obtain a homogeneous film on the wall of the vial. Any trace of the solvent was then removed, keeping the vial under vacuum for 15 min. Then, the dry lipid film was hydrated with 1.0 mL of 100 mM PBS and sonicated in an ultrasound bath for 30 min. The liposomal suspension (at a concentration of 800 μM) was extruded 10 times at RT, using a thermobarrel extruder system (Northern Lipids Inc., Vancouver, BC, Canada) under nitrogen through a polycarbonate membrane (Nucleopore Track Membrane 25 mm, Whatman, Brentford, UK) having 0.1 μm pore size. Lipoplex was prepared by adding 500 μL of a siRNA solution (5.2 μM in 0.1 mM buffer) to 500 μL of the liposome suspension. The resulting mixture was stirred at RT for 3 h. Successively, lipoplex suspension was shared in two vials: the first aliquot was diluted with an equal amount of buffer in order to obtain a final siRNA concentration of 1.3 μM (untargeted lipoplex); the second aliquot was functionalized with Ritux_Fab_ or Ritux_Fab_/FITC-Ritux_Fab_ (90/10, mol/mol) mixture (targeted lipoplex). 0.1 equivalents of Ritux_Fab_ (~0.5 mg/mL) with respect to the maleimide function were added to the liposomal suspension and the reaction was left ON. The unreacted Ritux_Fab_ was removed from the lipoplex by using Sepharose CL-4B column, pre-equilibrated with 0.1 M PBS. Nude liposomes were functionalized with Ritux_Fab_ according to the same procedure used for lipoplex. Liposome formulations for cytofluorimetric analysis were prepared following the same procedure described above, adding the fluorescent phospholipid NBD-PE dye to the lipid solution, during the preparation of the lipid film.

### 4.6. DLS Measurements

Mean diameter of liposomes and lipoplexes before and after functionalization with Ritux_Fab_ was measured by DLS using a Zetasizer Nano ZS 326 (Malvern Instruments, Westborough, MA, USA). Instrumental settings for the measurements are a backscatter detector at 173° in automatic modality, temperature of 25 °C and disposable sizing cuvette as cell. DLS measurements in triplicate were carried out on aqueous samples after centrifugation at RT at 13,000 rpm for 5 min.

### 4.7. siRNA Design

Authentic siRNA sequences were designed to specifically target C1858T *PTPN22* gene variant (Rosetta Inpharmatics, Sigma-Aldrich, Saint Louis, MO, USA). The siRNA sequences, sense/antisense (s/a) duplexes were different for mRNA target affinity and did not comprise backbone modifications as previously reported (Italian Patent 102018000005182 released on 26 June 2020. PCT/IT2019/050095 filed on 8 May 2019) [6].

### 4.8. Cell Culture

PBMC were separated by Ficoll–Hypaque (Histopaque, Sigma-Aldrich, Milan, Italy) from sodium heparinized venous blood samples (5–10 mL) belonging to recruited T1D patients and HD. Subsequently, PBMC were frozen in liquid nitrogen according to standard protocols [31]. The Raji human B lymphocyte cell line (Burkitt’s lymphoma) was obtained from American Type Culture Collection (ATCC CCL-86). Liquid nitrogen frozen cells were thawed and grown in RPMI-1640 Medium (Gibco^TM^, ThermoFisher Scientific, Waltham, MA, USA) supplemented with 10% fetal bovine serum (FBS, GE Healthcare Life Sciences, Logan, UT, USA), 2 mM L-glutamine (EuroClone S.p.A., Milan, Italy) and 1% penicillin/streptomycin (pen/strep) (EuroClone) according to established protocols [41]. Cells were incubated at 37 °C and in 5% CO_2_ at a density of between 0.1 and 1 × 10^6^/mL. Cells viability was determined by trypan blue dye staining. Cultures with more than 90% cell viability were used for the experiments.

### 4.9. FACS Analysis

#### 4.9.1. Cytofluorimetric Analysis of Raji Cell Line and Human PBMC

Cryopreserved PBMC were quickly thawed in pre-warmed RPMI-1640 Medium (Gibco^TM^) supplemented with 10% FBS (GE Healthcare Life Sciences), 2 mM L-glutamine (EuroClone) and 1% pen/strep (EuroClone) and centrifuged at 1200 rpm for 5 min at RT according to established protocols [31]. Cell pellets of T1D patients, HD and Raji cell line of approximately 5 × 10^5^ cells were allocated for each staining condition.

In two different subsequent experiments (*vide infra*) of binding analysis, cells were stained with FITC-Ritux_Fab_ and liposomal suspensions composed of Ritux_Fab_/FITC-Ritux_Fab_ (90/10 mol/mol). Then, the binding of FITC labelled lipoplexes functionalized with Ritux_Fab_ was verified.

#### 4.9.2. Binding of Ritux_Fab_ and the Liposomal Suspensions Derivatized with Ritux_Fab_/FITC-RituxF_ab_ (90/10 mol/mol)

Cell pellets of Raji cells and human PBMC were incubated with a directly conjugated mouse anti-human MoAb CD19- Brilliant Ultraviolet 737 (BUV737, Clone SJ25C1; BD Biosciences, San Jose, CA, USA) at 1:20 dilution for 20 min at 4 °C in the dark. Cells were then washed in 500 µL of PBS containing 2% FBS (FACS medium, EuroClone) by centrifugation at 1200 rpm for 5 min, then incubated with 20 µL of Fab(CD20)-FITC or the mixture of liposomes decorated with Ritux_Fab_/FITC-Ritux_Fab_ (90/10) for 20 min at 4 °C in the dark. After incubation, the non-binding lipoplexes were aspirated carefully by fine needle, then cells were washed in 500 µL of FACS medium by centrifugation at 1200 rpm for 5 min. Data were acquired using the BD LSR Fortessa X-20 flow-cytometer (Becton and Dickinson (BD Biosciences) and analyzed by using FACSDiva software (BD Biosciences). Dead cells were excluded from analysis by side/forward scatter (SSC/FSC) gating. A minimum of fifty thousand gated events on living cells were collected per dataset.

#### 4.9.3. Binding Assays of Ritux_Fab_-Lipoplexes

Cell pellets of Raji cells and human PBMC were incubated with 50 µL of either lipoplexes or Ritux_Fab_-Lipoplexes labelled with FITC for 30 min at 4 °C in the dark. To further unravel that the functionalization enriches the binding of lipoplexes to B lymphocytes, the same pellets were also incubated with lipoplexes labelled with the lipophilic fluorophore NBD-PE located in the inner lipid bilayer. In detail, 50 µL of NBD-PE labelled lipoplexes were incubated for 30 and 60 min at 4 °C in the dark. In both procedures after incubation, the non-binding lipoplexes were aspirated carefully by fine needle and then the cells were washed in 500 µL of FACS medium by centrifugation at 1200 rpm for 5 min.

### 4.10. Confocal Microscopy Analysis of Ritux_Fab_ Binding to Human B Lymphocytes within PBMC

Cell pellets of PBMC from one healthy donor were incubated with 20 µL of FITC-Ritux_Fab_ (2 mg/mL) for 30 min at 4 °C in the dark. After the incubation, the non-binding lipoplexes were aspirated by fine needle and then the cells were washed in 1 mL of PBS buffer for two times by centrifugation at 700 rpm for 5 min and fixed with paraformaldehyde 4% (*w*/*v*) (Sigma-Aldrich) for 10 min. Fixed cell suspensions were distributed drop wise onto positive charged microscope slides (Super Frost plus, Menzel-Glaser, Germany) and dried at 37 °C. After rehydration in PBS, cells were incubated with blocking solution PBS supplemented with bovine serum albumin (Sigma-Aldrich) 5% (*w*/*v*) for 30 min at RT.

Cells were subsequently incubated with primary mouse anti-human MoAb CD3 BV510 (Clone UCHT1, 1:30 dilution, BD Biosciences) for 60 min at RT or with primary mouse anti-human MoAb CD19 (Clone HIB19, 1:10 dilution, BD Biosciences) ON at 4 °C followed, after washing, by goat anti-mouse AlexaFluor 647 secondary antibody (1:400 dilution, ThermoFisher Scientific) for 1 h at RT. Another set of cells was double stained by combining the same antibodies. After washing with PBS, cells were incubated with SYTO™82 Orange Fluorescent dye (1:1000 dilution, ThermoFisher Scientific) for 10 min at RT to counterstain nuclei. Thereafter, cells were washed with PBS and mounted in PBS 40%/glycerol 60% solution and cover-slipped before being evaluated by Leica TCS AOBS-SP8X laser-scanning confocal microscope (Leica Microsystems, Mannheim, Germany) equipped with tunable white light laser (WLL, 470–670 nm of wavelength) source, 405 nm diode laser, 3 PMT, 2 HyD detectors and an acousto-optical beam splitter (AOBS) that allowed the separation of multiple fluorescences, using a 63× (1.42 NA oil) objective. Optical single sections were acquired with a scanning mode format of 512 × 512 pixels, sampling speed of 400 Hz (pixel size of 0.103 μm), and Z-reconstructions of serial single optical sections were carried out with an electronic zoom at 3.5. Fluorochromes unmixing was performed by acquisition of automated-sequential collection of multi-channel images, in order to reduce spectral crosstalk between channels.

### 4.11. Functional Assay on CpG—Stimulated B Lymphocytes

In order to verify the functional effect of Fab functionalized lipoplexes, we tested them on a functional assay in which PBMC of an autoimmune condition, that is, T1D were challenged with CpG oligodeoxynucleotides (Hycult Biotechnology, Uden, The Netherlands).

#### 4.11.1. Human Patients PBMC

PBMC of eight T1D patients heterozygous carriers of the C1858T *PTPN22* variant were recruited from the Endocrinology Division at Bambino Gesù Children’s Hospital. All patients were recruited during long-term disease. The mean age at onset of disease was 8.9 years (age range 2–12.7), and the mean disease duration was 9.8 years (age range 4.4–19.2). The mean age at referral of the patients was 18.7 years (age range 11.6–31.1; two females, six males) (Table 1). Diabetics’ demographic and clinical characteristics are shown in Table 1. The patients’ sera were assayed for glutamic acid decarboxylase (GADA, isoform 65), protein tyrosine phosphatase insulinoma-associated antigen 2 (IA2) and anti-insulin (IAA) antibodies (Abs) by radioimmunoassay, for Abs to thyroglobulin (Tg), thyroperoxidase (TPO) and transglutaminase (tTGA) by chemiluminescence (ADVIA Centaur analyzer, Siemens Healthcare, Germany) and to parietal cells (PCA) and adrenal cortex by indirect immunofluorescence (IFL). The T1D patients presented associated autoimmune disorders, that is, autoimmune thyroid disease (Graves’ disease) (patient number (N°) 4) and vitiligo (patient N° 8) (Table 1).

All recruited patients were unrelated. All subjects entered the study after written informed consent was obtained. The investigation was approved by the local Institutional Review Board (IRB) of Bambino Gesù Children’s Hospital, which regulates human samples usage for experimental studies (Study protocol no. RF-2019-12369889); all procedures followed were in accordance with institutional guidelines. The informed consent was obtained from the next of kin in case of children. Consent on behalf of children was written. Participant consent was recorded using a paper-based inventory system. The IRB approved the consent procedure.

#### 4.11.2. Stimulation of PBMC with CpG and Proliferation Assay

Liquid-nitrogen frozen PBMC from T1D patients were quickly thawed as previously described [31]. Cells were centrifuged at 1200 rpm for 5 min at RT and seeded in 48-well plates (flat bottom, Falcon, Corning, NY, USA) at a density of 1.5 × 10^6^ per well in a final volume of 250 μL of FBS-free RPMI 1640 medium containing L-glutamine (2 mM) and treated with different doses of lipoplex and Ritux_Fab_-Lipoplex (80 and 100 pmols of siRNA). After approximately 16 h (ON) of transfection, cells were washed in complete RPMI by 1200 rpm centrifugation for 5 min at RT.

Before stimulation, PBMC were labeled with 5-chloromethylfluorescein diacetate (CMFDA) (CellTracker, Invitrogen, Molecular Probes, Eugene, OR, USA) at a final concentration of 0.1 mg/mL and cultured at 7.5 × 10^5^ cells per well in 96-well plates (Falcon, Labware BD Biosciences, Oxnard, CA, USA) in complete RPMI 1640 medium supplemented with 10% FBS (Hyclone, South Logan, UT, USA), L-glutamine (2 mM) and 1% pen/strep. The cells were stimulated with human CpG at a concentration of 5 mg/mL and supplemented with IL-2 (25 IU/mL, Sigma Aldrich) according to established protocol [31]. IL-2 was added to the cultures because we found that it improved cell survival in cryopreserved pathological samples without altering cell function. The cells were incubated for 4 days at 37 °C in a humidified atmosphere containing 5% CO_2_. In parallel, basal PBMC cultures were set up by incubating PBMC from the same individual with complete medium plus IL-2 as a control. After 4 days of CpG stimulation, PBMC were harvested from the culture plates and washed by centrifugation at 1200 rpm for 5 min at RT in FACS medium. To identify B cell subsets, single-cell suspensions were stained with the appropriate combination of the following directly conjugated MoAbs: CD19-BUV 737 (1:20 dilution; BD Biosciences), CD38-PECy7 (1:90 dilution; BD Biosciences), CD27-PE (1:20 dilution; BD Biosciences) and IgM-Alexa Fluor 647 (1:400 dilution; Jackson ImmunoResearch, West Baltimore Pike, PA, USA). Cells were incubated for 20 min in the dark at 4 °C. After labelling, cells were washed by centrifugation at 1200 rpm for 5 min at 4 °C in FACS medium. Data were acquired using BD LSR Fortessa X-20 flow-cytometer (BD Biosciences) and analyzed by using FACSDiva software (BD Biosciences). Dead cells were excluded from the analysis by side/forward scatter gating. A minimum of fifty thousand gated events on living cells were collected per dataset.

### 4.12. Statistical Analysis

Values are expressed as means ± SEM. Differences between each test condition were assessed by one-way ANOVA followed by Bonferroni and Dunnett’s multiple comparison tests, for the functional assay on T1D PBMC. Differences between CD19+/Ritux_Fab_ and CD19+/FITC-Ritux_Fab_ cells were assessed by the unpaired Mann–Whitney *t*-test, for the binding of FITC-Ritux_Fab_ functionalized liposome to Raji cells and lipoplexes functionalized with Ritux_Fab_/FITC-Ritux_Fab_ (90/10 mol/mol) to HD PBMC experiments. Differences between CD19+/Lipoplex-NBD PE+ and CD19+/Ritux_Fab_-Lipoplex-NBD PE+ cells were determined by the unpaired Mann–Whitney *t*-test, for the binding of functionalized NBD PE-lipoplexes to Raji cells and human B lymphocytes within the PBMC pool. The statistical study was performed with GraphPad Prism (version 7, San Diego, CA, USA). The difference was considered statistically significant at *p*  < 0.05.

## 5. Patents

Italian Patent 102018000005182 released on 26 June 2020. PCT/IT2019/050095 filed on 8 May 2019. Inventor: Dr. Alessandra Fierabracci. Property: Bambino Gesù Children’s Hospital, IRCCS, Rome, Italy.

## Figures and Tables

**Table 1:** Demographic, genetic and clinical characteristics of patients of the present study.

Patient	Gender	Age of Disease Onset (Years)	Age at Referral (Years)	Duration of Disease (Years)	Autoimm-ne Disorders	*PTPN22* Genotype	Islet Related AABs	Other AABs
1	M	11.1	19.4	8.3	T1D	1858C/1858T	GADA **5.3 U/mL**; IAA **11%**; IA2 **29 U/mL**	tTGA 0.2 U/mL; TPO 40.5 U/mL; Tg < 20.0 UI/mL
2	M	8.6	18.1	9.5	T1D	1858C/1858T	GADA 0.3 U/mL; IAA **8%**; IA2 **2.0 U/mL**	tTGA 0.2 U/mL; TPO 40.3 U/mL; Tg 17.3 U/mL
3	M	10.5	18	7.5	T1D	1858C/1858T	GADA **16 U/mL**; IAA 4.1%; IA2 **2.0 U/mL**	tTGA 0.2 U/mL; TPO < 28.0 U/mL; Tg < 20.0 UI/mL
4	M	11.9	31.1	19.2	Graves’ disease T1D	1858C/1858T		tTGA 0.4 U/mL; TPO > **1300 U/mL**; Tg > **2500 U/mL**;
5	M	12.7	18.9	6.3	T1D	1858C/1858T	GADA **5.3 U/mL**; IAA **22%**; IA2 **2.3 U/mL**	tTGA < 1.9 CU; TPO < 28.0 U/mL; Tg < 20.0 UI/mL
6	F	2	15.8	13.7	T1D	1858C/1858T	GADA **18.6 U/mL**; IAA **67.0%;** IA2 0.1 U/mL	tTGA 0.20 U/mL; TPO 19.9 U/mL; Tg 15.2 U/mL
7	M	7.1	11.6	4.4	T1D	1858C/1858T	GADA**12.2 U/mL**; IAA **7.4%**; IA2 **2.7 U/mL**	tTGA 1.9 CU; TPO 30.11 U/mL; Tg < 20.0 UI/mL
8	F	7.2	16.7	9.5	T1D Vitiligo	1858C/1858T	GADA **1.8 U/mL**; IAA 2.4%; IA2 **15 U/mL**	tTGA 0.2 U/mL; TPO 35.9 U/mL; Tg < 20.0 UI/mL

## Data Availability

The original contributions presented in the study are included in the article/Supplementary Materials; further inquiries can be directed to the corresponding author.

## Supplementary Materials

The following supporting information can be downloaded at: https://www.mdpi.com/article/10.3390/ijms23010408/s1.
