# Supplementary material for: Preparation and In Vitro Evaluation of RITUXfab-Decorated Lipoplexes to Improve Delivery of siRNA Targeting C1858T PTPN22 Variant in B Lymphocytes
Source: Int J Mol Sci. 2021 Dec 30;23(1):408. doi: 10.3390/ijms23010408 (PMC8745767; doi:10.3390/ijms23010408)
Supplement: Supplementary file 1 [file ijms-23-00408-s001.zip › ijms-1513691-supplementary.pdf]

Figure S1

A

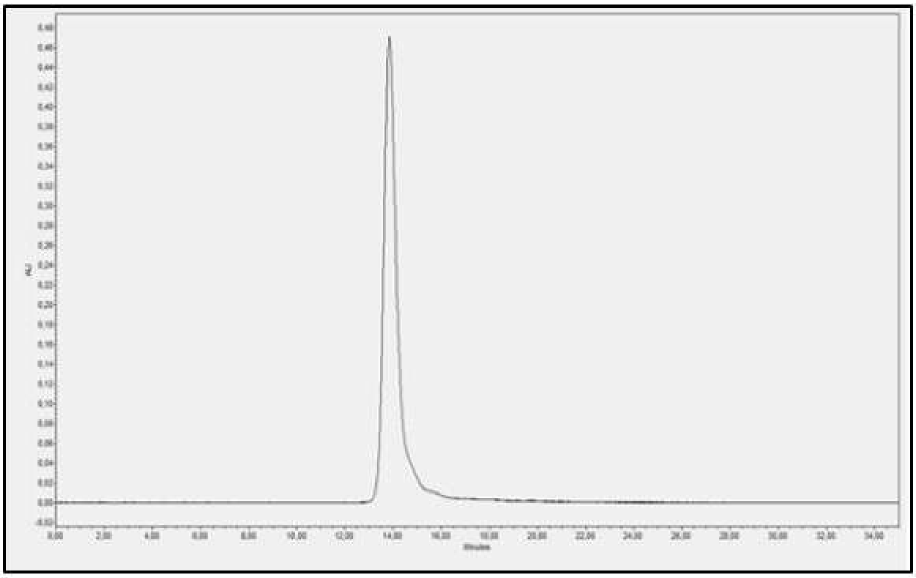

B

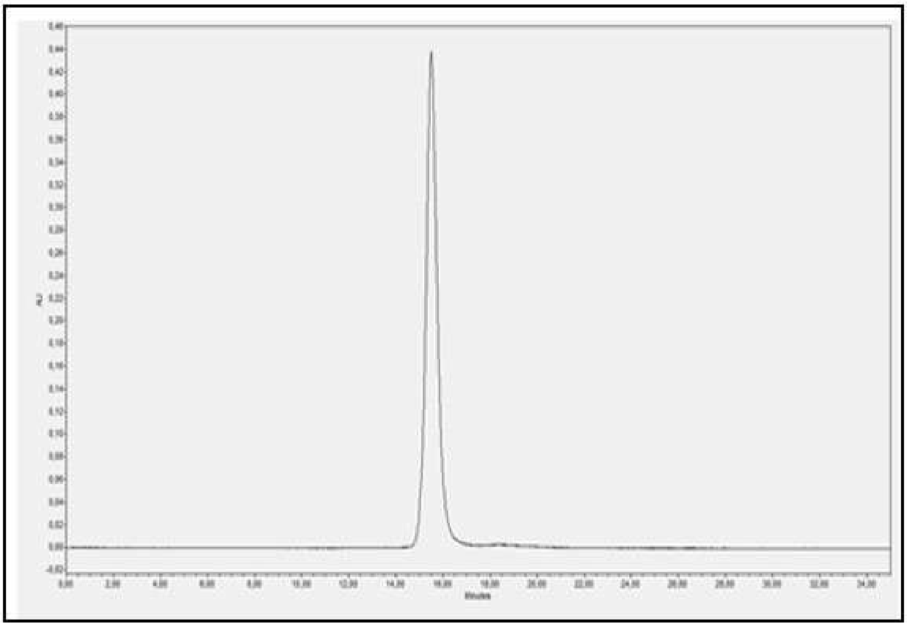

Figure S2

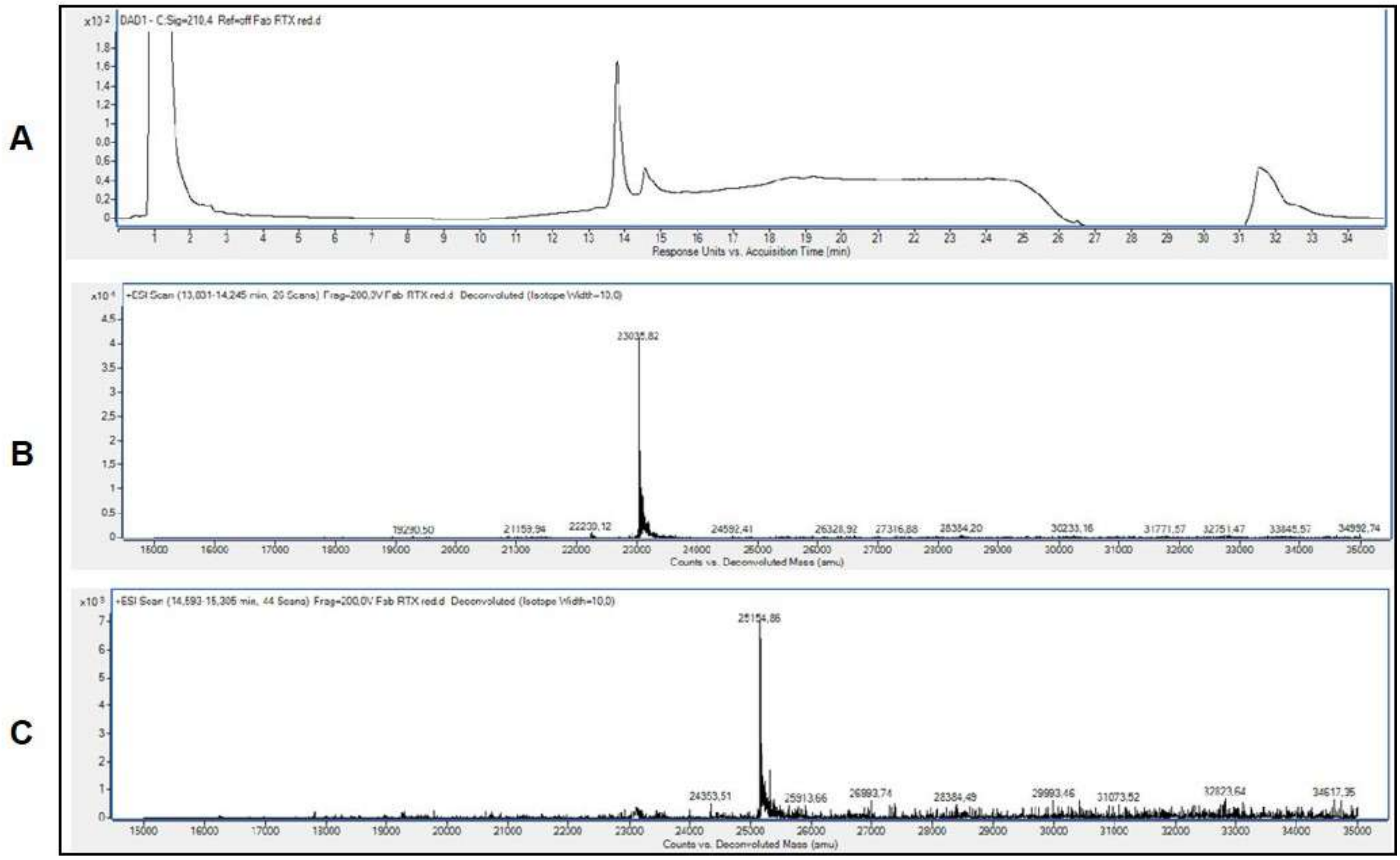
A
DAD1 - C:Sig=210,4 Ref=off Fab RTX red.d
Response Units vs. Acquisition Time (min)
B
+ESI Scan (13,031-14,245 min, 20 Scans) Frag=200,0V Fab RTX red.d Deconvoluted (Isotope Width=10,0)
23035,82
19290,50
21159,94
22230,12
24592,41
26328,92
27316,88
28384,20
30233,16
31771,57
32751,47
33845,57
34992,74
Counts vs. Deconvoluted Mass (amu)
C
+ESI Scan (14,593-15,306 min, 44 Scans) Frag=200,0V Fab RTX red.d Deconvoluted (Isotope Width=10,0)
25154,86
24353,51
25913,66
26993,74
28384,49
29993,46
31073,52
32823,64
34617,35
Counts vs. Deconvoluted Mass (amu)

Figure S3

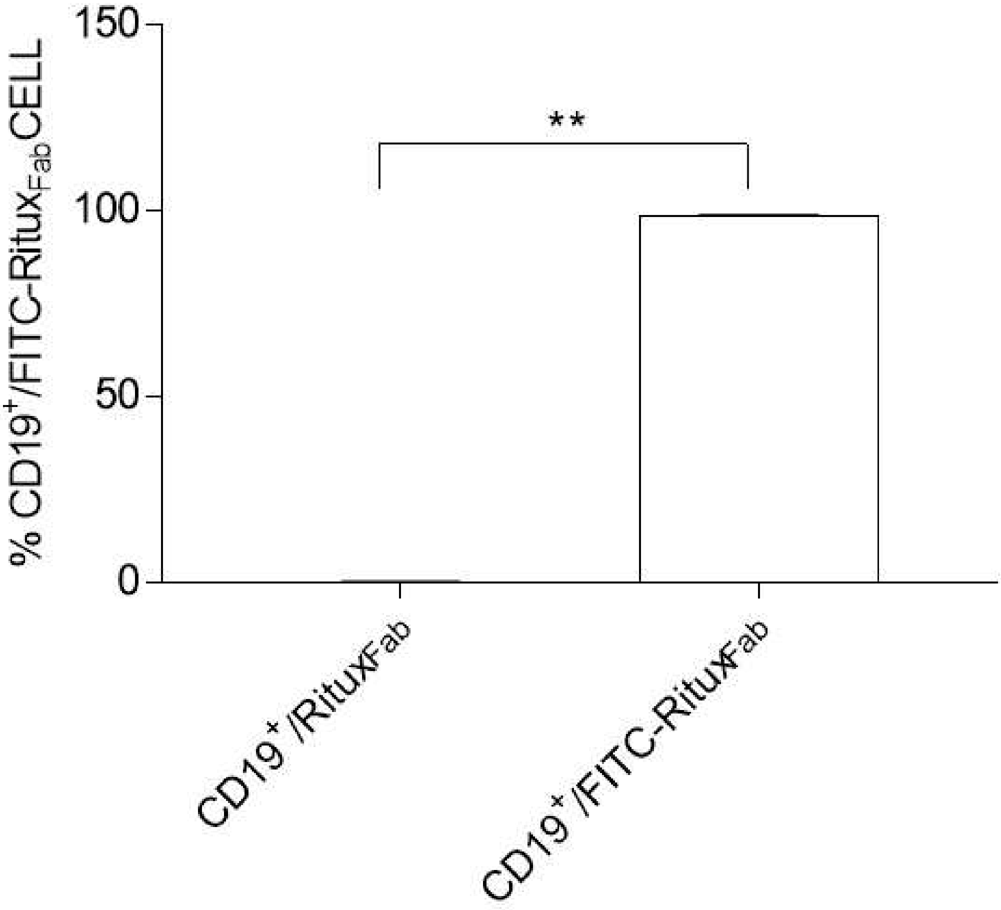

150
100
50
0
**
% CD19+/FITC-RituxFab CELL
CD19+/RituxFab
CD19+/FITC-RituxFab

Figure S4

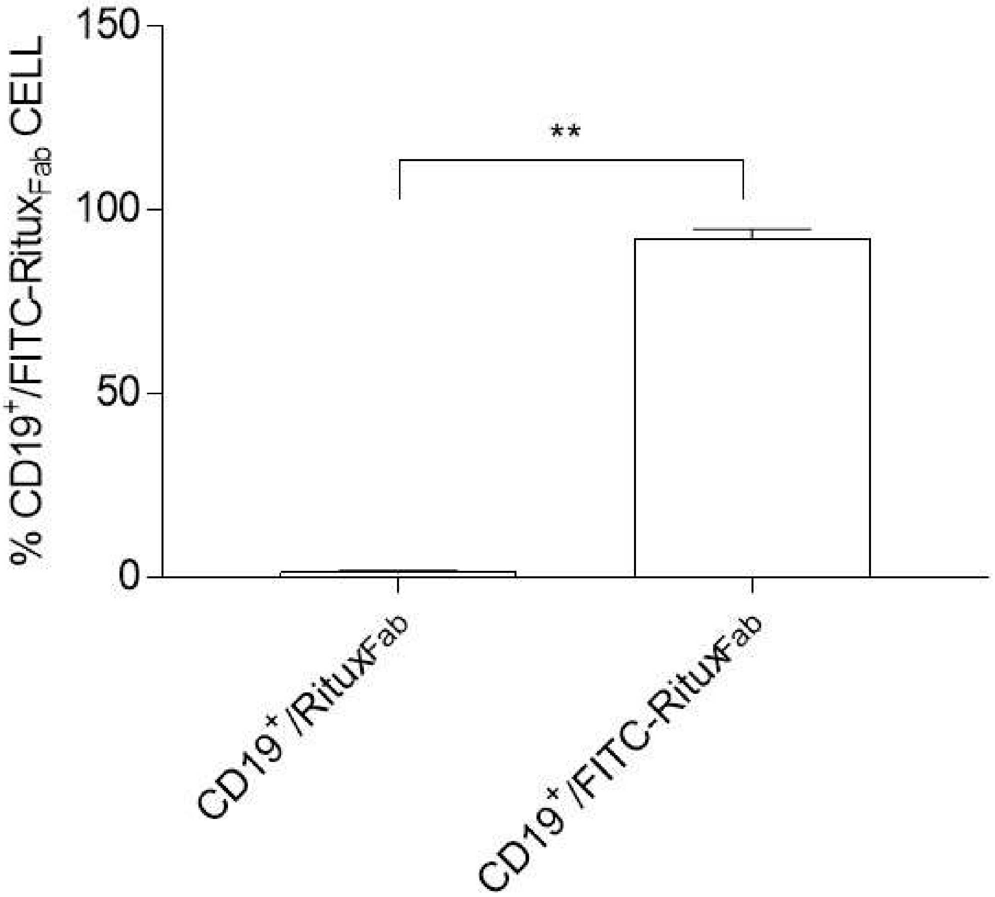
% CD19+/FITC-RituxFab CELL
150
100
50
0
**
CD19+/RituxFab
CD19+/FITC-RituxFab

Figure S5

A

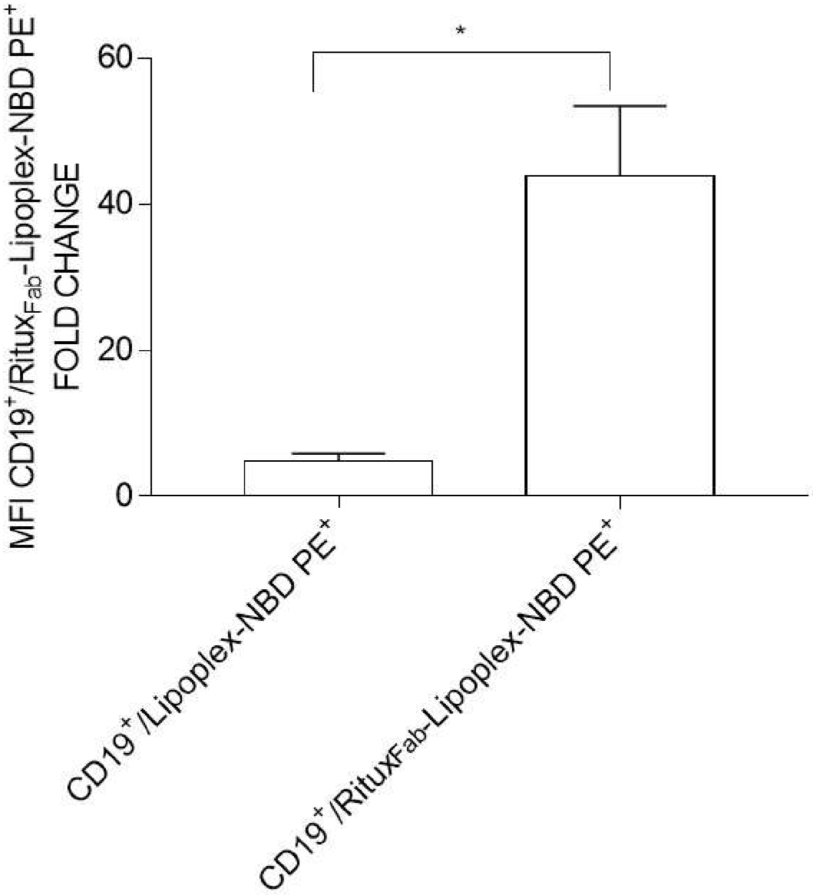

MFI CD19+/RituxFab-Lipoplex-NBD PE+
FOLD CHANGE
60
40
20
0
*
CD19+/Lipoplex-NBD PE+
CD19+/RituxFab-Lipoplex-NBD PE+

B

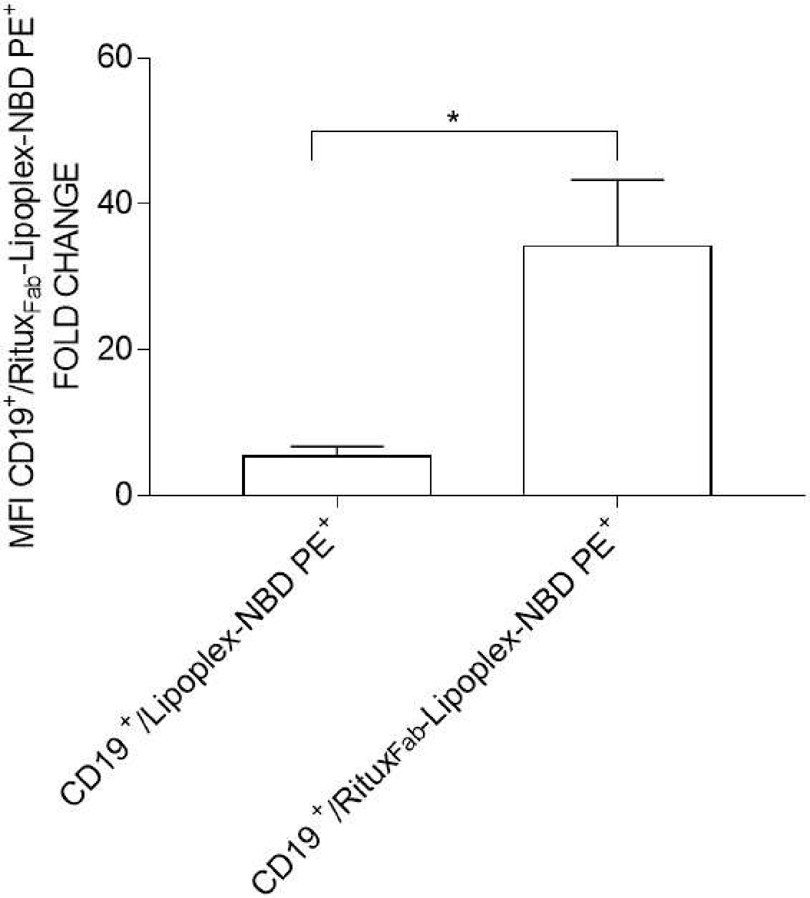

MFI CD19+/RituxFab-Lipoplex-NBD PE+
FOLD CHANGE
60
40
20
0
*
CD19+/Lipoplex-NBD PE+
CD19+/RituxFab-Lipoplex-NBD PE+

Figure S6

A

B

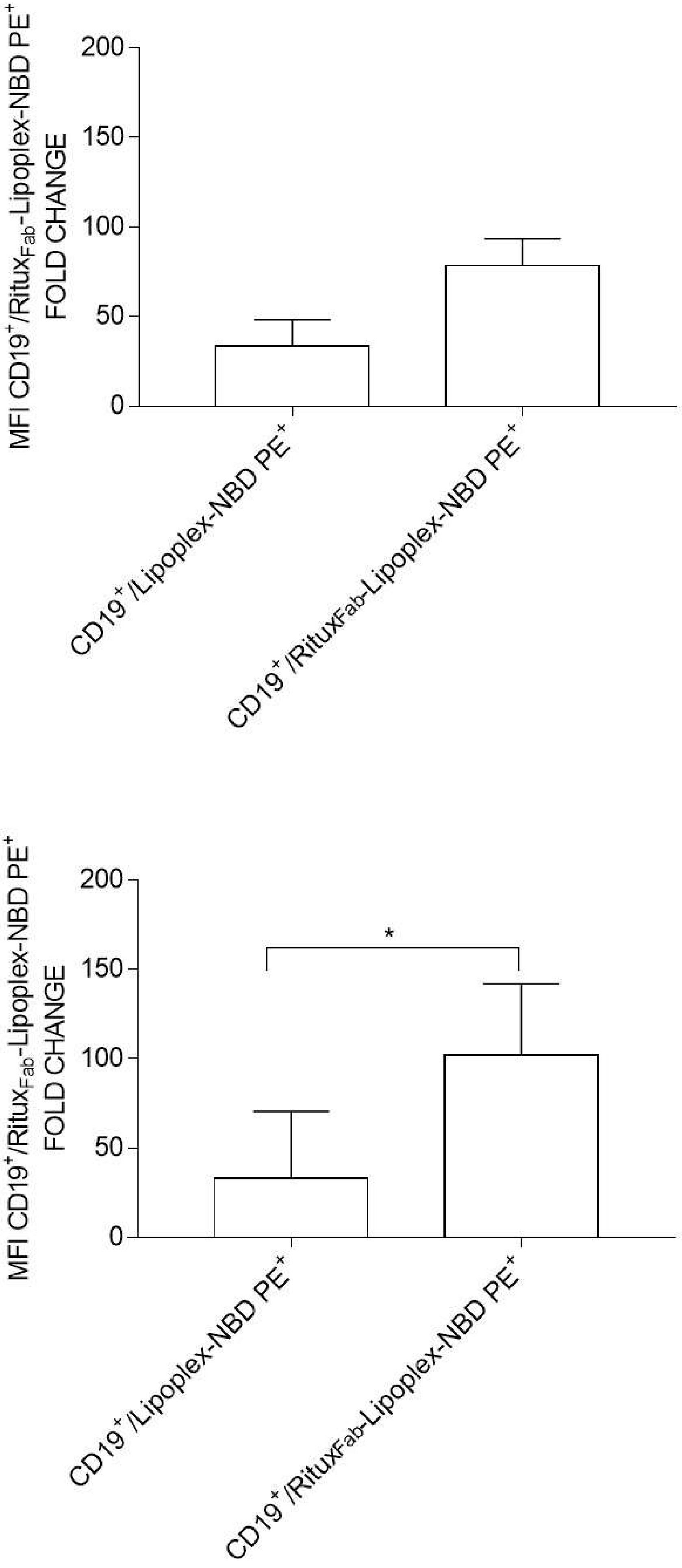

MFI CD19+/RituxFab-Lipoplex-NBD PE+
FOLD CHANGE
200
150
100
50
0
CD19+/Lipoplex-NBD PE+
CD19+/RituxFab-Lipoplex-NBD PE+
MFI CD19+/RituxFab-Lipoplex-NBD PE+
FOLD CHANGE
200
150
100
50
0
*
CD19+/Lipoplex-NBD PE+
CD19+/RituxFab-Lipoplex-NBD PE+

Supplementary Figure 1. Analytical SEC analysis. SEC-HPLC analytical characterization of purified RituxFab2 (CD20) (Rt 14 min) (**A**) and RituxFab (CD20) (Rt 16 min) (**B**). The analyses were performed on a BioSep-SEC 2000 column at a flow rate of 0.6 mL/min.

Supplementary Figure 2. LC-ESI-TOF-MS analysis of the reduced $Ritux_{Fab}$. RP-HPLC profile showing the separated LC (peak at 13.9 min) and HC (peak at 14.8 min) (**A**). Deconvoluted mass charge spectrum of the LC (**B**). Deconvoluted mass charge spectrum of the HC (**C**).

Supplementary Figure 3. Binding of FITC-$Ritux_{Fab}$ to Raji cell line. Differences between CD19+/$Ritux_{Fab}$ and CD19+/FITC-$Ritux_{Fab}$ cells after 30 min of incubation were statistically evaluated using the unpaired Mann Whitney t-test. Data are expressed as mean ± SEM of n = 5. * p<0.05.

Supplementary Figure 4. Binding of FITC-$Ritux_{Fab}$ to HD PBMC. Differences between CD19+/$Ritux_{Fab}$ and CD19+/FITC-$Ritux_{Fab}$ cells after 30 min of incubation were statistically evaluated using the unpaired Mann Whitney test t-test. Data are expressed as mean ± SEM of n = 5. * p<0.05.

Supplementary Figure 5. Evaluation of the specific binding of $Ritux_{Fab}$-Lipoplexes labelled with the NBD PE fluorescent dye to Raji cell line. Differences between CD19+/Lipoplex-NBD PE+ and CD19+/$Ritux_{Fab}$ Lipoplex-NBD PE+ cells after 30 min (**A**) or 60 min (**B**) of incubation were assessed for statistical significance with the unpaired Mann Whitney test t-test. Data are expressed as mean ± SEM of n = 3. * p<0.05.

Supplementary Figure 6. Evaluation of the specific binding of $Ritux_{Fab}$-Lipoplexes labelled with the NBD PE fluorescent dye to HD PBMC. Differences between CD19+/Lipoplex-NBD PE+ and CD19+/$Ritux_{Fab}$-Lipoplex-NBD PE+ cells after 30 min (**A**) or 60 min (**B**) of incubation were assessed for statistical significance with the unpaired Mann Whitney test t-test. Data are expressed as mean ± SEM of n = 5. * p<0.05.
